# The Clinical Teaching Fellow role: exploring expectations and experiences

**DOI:** 10.1186/s12909-024-05207-6

**Published:** 2024-03-01

**Authors:** Isobel Marion Harris, Heather McNeilly, Derek J. Ward, Alice J. Sitch, Jayne Parry, Sheila Greenfield

**Affiliations:** 1 Institute of Applied Health Research, University of Birmingham, Birmingham, UK; 2 Institute of Clinical Sciences, University of Birmingham, Birmingham, UK; 3 NIHR Birmingham Biomedical Research Centre, University Hospitals Birmingham NHS Foundation Trust and University of Birmingham, Birmingham, UK

**Keywords:** Teaching fellows, Undergraduate medical education, Workforce, Teaching

## Abstract

**Background:**

Many UK junior doctors are now taking a year out of the traditional training pathway, usually before specialty training, and some choose to work as a clinical teaching fellow (CTF). CTFs primarily have responsibility for delivering hospital-based teaching to undergraduate medical students. Only a very small amount of literature is available regarding CTF posts, none of which has explored why doctors choose to undertake the role and their expectations of the job. This study aimed to explore the expectations and experiences of CTFs employed at NHS hospital Trusts in the West Midlands.

**Methods:**

CTFs working in Trusts in the West Midlands region registered as students on the Education for Healthcare Professionals Post Graduate Certificate course at the University of Birmingham in August 2019 took part in a survey and a focus group.

**Results:**

Twenty-eight CTFs participated in the survey and ten participated in the focus group. In the survey, participants reported choosing a CTF role due to an interest in teaching, wanting time out of training, and being unsure of which specialty to choose. Expectations for the year in post were directly related to reasons for choosing the role with participants expecting to develop teaching skills, and have a break from usual clinical work and rotations. The focus group identified five main themes relating to experiences starting their job, time pressures and challenges faced in post, how CTF jobs differed between Trusts, and future career plans. Broadly, participants reported enjoying their year in a post at a mid-year point but identified particular challenges such as difficulties in starting the role and facing time pressures in their day-to-day work.

**Conclusion:**

This study has provided a valuable insight into the CTF role and why doctors choose a CTF post and some of the challenges experienced, adding to the sparse amount of literature. Understanding post holders’ experiences may contribute to optimisation of the role. Those employing CTFs should consider ensuring a formal handover process is in place between outgoing and incoming CTFs, having a lead person at their Trust responsible for evaluating changes suggested by CTFs, and the balance of contractual duties and personal development time.

**Supplementary Information:**

The online version contains supplementary material available at 10.1186/s12909-024-05207-6.

## Introduction

In order to address concerns about understaffing in the NHS and increasing demand on hospital services, the number of medical school places in the UK has been expanded and it is thought that further expansion will be required to ensure sustainability [1, 2]. With clinicians already facing increased demand to deliver teaching [3–6], an expansion in the number of medical students will further increase this need for teaching in a clinical setting. One way the demand for teaching is being addressed is through the creation of the Clinical Teaching Fellow (CTF) role.

CTFs are junior doctors who have taken time out of the usual training pathway to be employed in a role that has responsibility for undergraduate medical education and which delivers teaching that was traditionally delivered by more senior hospital doctors. CTF roles mostly have a one year contract and based in hospitals [7], although a small number of posts may be based at universities [8]. There are differences between CTF posts with the roles varying in terms of the split between teaching and clinical work, as well as other duties including research, and student pastoral support [7, 9–11]. Post-holders also usually have the opportunity to obtain postgraduate qualifications related to education offered alongside the role [7]. Most published literature regarding CTFs describes the role in a UK setting, but similar posts do exist in other countries [12].

It is not known exactly how many CTFs are employed across the UK, but the role has been increasing in numbers over recent years. A study in 2005 recorded 77 CTF posts across the UK [11], but a study in 2018 reported 101 posts in one geographical area of the UK alone (North East England), with 26 of those employed at a single hospital Trust [10].

CTFs are a population of doctors in whom only limited research has previously been carried out. The current available literature is primarily career advice/opinion pieces [7, 13–18] which are usually written by previous CTFs for doctors who may be considering applying for a CTF post. A very small number of primary studies exist which have attempted to map out the CTF role [11], evaluate the CTF role from the point of view of undergraduate medical students [19], and we have previously explored the views of senior hospital doctors in the West Midlands regarding CTFs [20]. Currently there is no literature exploring why doctors choose to work in a CTF role or what their expectations of the role are, and there is only one study available (Ker et al [10]) that has looked at the challenges of the post from the perspective of the CTFs themselves. Ker et al. recruited CTFs, as well as other related stakeholders including administrative staff and consultants with a role in education, to attend a one-off workshop at a medical education conference in 2018 to discuss common challenges facing CTFs. Ker et al.’s study identified seven key areas of concerns for the participants: issues with role identity, different aspects of the role, continuity of changes made in the role, pastoral support expectations, role stigma, being used to fill rotation gaps, and lack of further career support. Whilst this study has provided insights into the challenges facing CTFs, the authors acknowledged that further research is required to deepen understanding of these issues.

With no literature exploring expectations and experiences of CTFs currently available and an increasing number of CTF posts, an understanding of why doctors choose a CTF post, their expectations of the post, and the perspectives of those in post will be beneficial for the post holders themselves, NHS Trusts employing them, and those responsible for policy decisions regarding medical education to ensure maximum benefit can be derived from the role. This paper reports the first part of a longitudinal study exploring the experiences and future career plans of CTFs in the West Midlands which aimed to explore the expectations of CTFs prior to starting their role.

## Methods

To explore expectations and experiences a qualitative longitudinal research methodological approach was used; a survey was firstly administered at the beginning of their year, followed by a focus group mid-way through the year. Following the survey with a focus group allowed for deeper exploration of the results of the survey and the opportunity for participants to reflect upon their expectations of the role at a later time point. Focus groups are useful for exploring people’s experiences through group interaction [21], and in this case allowed the CTF participants to compare and contrast their own experiences in different hospital settings. Focus groups are an appropriate data collection methodology for this type of exploratory and phenomenological research as they are known to be of use in early stages of research to identify issues that are important to participants and the findings can then be used to inform future work [22].

## Setting

This study took place at the University of Birmingham (UoB).

### Participants

#### Baseline survey

All CTFs employed by and working in NHS Trusts (organisational units of the NHS providing health services) in the West Midlands region where UoB medical students undertake placements are offered the opportunity to complete the Education for Healthcare Professionals Post Graduate Certificate (PGCert) course at UoB during their year in post. Student registration on the PGCert course offered a convenient sampling frame to identify the CTFs teaching UoB medical students for we assumed that the majority of CTFs starting their role would not already have a PGCert qualification and therefore would be taking up the offer to register on the programme. Therefore, all CTFs working in Trusts in the West Midlands region where UoB medical students undertake placements who were registered as students on the PGCert course at UoB in August 2019 were invited to take part in this study.

#### Focus group

All participants who had completed the baseline survey were invited by email to take part in a focus group to discuss their responses to the baseline survey. Two reminder emails were sent to participants who did not respond. Participants who did respond were emailed the Participant Information Sheet which explained the study and how data would be handled and a consent form to fill in ahead of the focus group.

### Data collection

#### Baseline survey

Baseline surveys (Appendix 1) were distributed to participants electronically as a Word document (via email) or in hard copy paper format. The baseline survey contained questions to collect information on CTFs’ demographics, undergraduate/postgraduate medical training, reasons for choosing a CTF post, expectations for the year spent in post, and longer-term career plans. The survey was structured with a section of multiple choice questions at the beginning, and then two sections of topic-based questions (mostly free text answers).

The questions in the two topic-based sections of the survey sought to map out duties of the CTF role, as it is known there is variation in the responsibilities of a CTF post [10], and to explore perceived benefits and expectations of the role ahead of completing a post. These questions were based on our review of the limited literature detailing benefits of a CTF role [13, 14, 18], and discussion with colleagues. The survey was circulated to another member of staff at UoB who had previously held a CTF post to pilot it, no changes were made after this.

#### Focus group

The PGCert course involves several teaching days held in person at the university and therefore for convenience of the participants, the focus group was run in person at UoB during the lunchtime break in one of these teaching days in February 2020. A buffet lunch was provided as is usual for all students attending PGCert days so as not to disadvantage participants. The focus group took place in a separate location to the rooms used for their teaching and lasted for approximately 45 min.

IH (a white female in her thirties who is a doctoral researcher and Research Fellow in the Institute of Applied Health Research at UoB) gave a brief summary of the survey findings as a starting point for the discussion and then asked the participants what they thought about these and what their experiences of the post had been so far. The focus group followed a flexible topic guide (Appendix 2) with prompts loosely based on the questions asked in the baseline survey. These prompts covered expectations for the year, how the job matched these expectations, differences in CTF posts between different hospitals, and future career plans. IH acted as a facilitator for the focus group, asking initial and prompting questions where necessary, and encouraging discussion amongst the participants. This allowed for participants to discuss their own experiences in the context of others, and also allowed for a mid-year check as to how their expectations of the year had been met so far. A second researcher, HM, also a white female in her thirties who is employed at UoB as a clinical lecturer, was present to take notes but did not participate in the discussion, as recommended by Krueger [23]. The participants had previously met IH when she attended their earlier teaching days to introduce the study and to recruit to the survey.

### Data analysis

#### Baseline survey

Data from the surveys were entered into Microsoft Access and then exported into Microsoft Excel for initial analysis. Quantitative analysis of descriptive statistics from the survey was carried out by producing basic summaries from the demographic data. Thematic analysis was selected as the most appropriate method of data analysis to use for the free text answers in the survey as it is a method well suited for exploring views and experiences, and for identifying shared meanings across a dataset [24, 25]. The thematic analysis was conducted by following the approach described by Braun & Clarke [26], however, most of the answers given to the questions in the baseline survey were very short, consisting of single word or short sentence answers. Therefore, after carrying out the initial coding and categorisation of data, the decision was made to not continue with the further steps of thematic analysis that generate themes. Instead, a content analysis approach was taken where the number of participants that gave an answer in each category was counted, and this has been presented in a tabular form showing numbers and percentages of respondents giving an answer that had been coded and sorted into each category.

#### Focus group

The focus group was recorded, transcribed by IH, and analysed thematically following the approach described by Braun & Clarke [26]. The transcript was uploaded into NVivo 12 Plus [27] which was used to manage the data and carry out initial coding. After coding the transcript, the codes were exported into an *Excel* spreadsheet where related codes were sorted into columns to identify themes. The themes were reviewed by reading the coded extract attached to each one to decide whether they were in an appropriate column or would be better suited elsewhere. The transcript was then reread to assess whether the themes identified were reflective of the data. Once all the relevant data had been coded the themes were further refined and two sub themes were identified under one of the main themes. All data was coded by IH with regular meetings held with other members of the multidisciplinary research team (JP, Professor of Public Health, SG, Professor of Medical Sociology, DW, Reader in Public Health and Medical Education, AS, Senior Lecturer in Biostatistics) to discuss, refine, and agree the coding and analysis.

### Ethical considerations

Ethical approval was granted by UoB in May 2019 (ERN_19-0687). Subsequent amendments were made by chair decision (ERN_19-0687A) or approved in line with the UoB Research Ethics and Governance Exceptional Circumstances Due to COVID 19 guidance.

Informed consent was taken in accordance with the Good Clinical Practice guidelines using a consent form and included permission for the focus group to be digitally recorded and for anonymised quotations to be used in future reports or publications.

## Results

Out of the 44 CTFs registered on the PGCert course in 2019, 28 (63.6%) participated in the baseline survey. The 28 participating CTFs were based at 10 different hospitals. The ratio of male to female participants was 1:2, and the majority were taking up the CTF post immediately after two years of Foundation training (53.6%). A small proportion of participants had completed a prior degree to Medicine (14.3%), and approximately 40% had undertaken an intercalated degree.

Ten participants took part in the focus group (four male and six female) were based at six different Trusts, seven worked full time, and three worked part time. All had studied an undergraduate MBChB course and over half had undertaken an intercalated degree during their studies. The majority of participants had studied at a UK-based medical school with five having studied at UoB.

Participant characteristics are shown in Table 1.

**Table 1:** Participant characteristics for baseline survey and focus group

Characteristic	Survey [*N* = 28(%)]	Focus group [*N* = 10(%)]
Male/female		
*Male*	9 (32)	4 (40)
*Female*	19 (68)	6 (60)
Ethnicity		
*White*	15 (54)	6 (60)
*Mixed*	2 (7)	1 (10)
*Black*	3 (11)	1 (10)
*Asian*	4 (14)	1 (10)
*Other*	4 (14)	1(10)
MBChB course studied		
*Undergraduate*	25 (89)	10 (100)
*Graduate*	3 (10)	0 (0)
Location of medical school studied at		
*UK*	26 (93)	9 (90)
*University of Birmingham*	13/26 (50)	5/9 (56)
*Other UK medical school*	13/26 (50)	4/9 (44)
*Overseas*	2 (7)	1 (10)
Years after qualifying		
*Two years*	15 (54)	6 (60)
*Three-four years*	7 (25)	0 (0
*Five* + *years*	6 (21)	4 (40)
Prior degree	4 (14)	0 (0)
Intercalated degree	13 (46)	6 (60)
*Biosciences subject*	7/13 (54)	2/6 (33)
*Humanities subject*	6/13 (46)	4/6 (67)
Previous teaching qualification	3 (11)	1 (10)
Full time CTF post held	25 (89)	7 (70)
Part time CTF post held	3 (11)	3 (30)

### Baseline survey

All participants answered all of the questions.

#### “What will your key duties be?”

Whilst all participants described teaching medical students it appeared some posts were speciality specific, and others appeared to have responsibility for particular year groups of students (Table 2). Posts also varied in the amount of time dedicated to clinical duties, with the majority not mentioning any clinical work but others (*n* = 4) reporting up to 50% of time to be spent on clinical duties.

**Table 2:** Survey questions and responses

Question	N (%)
**What will your key duties be?**	
Teaching medical students	28 (100)
Simulation training	10 (36)
Teaching particular year group	10 (36)
Teaching other staff	5 (18)
Clinical duties (not teaching)	4 (14)
Organising/co-ordinating teaching	4 (14)
Supervisory/mentoring role for students	4 (14)
Teaching clinical skills	4 (14)
Teaching particular specialty	2 (7)
Running/organising assessments	1 (4)
**What do you think the role will involve?**	
Providing teaching	28 (100)
Planning/organising teaching	10 (36)
Simulation teaching	9 (32)
Supervisory/mentoring role for students	8 (29)
PGCert completion	5 (18)
Clinical duties (not teaching)	4 (14)
Running/helping with assessments	2 (7)
Research	1 (4)
Carrying out an audit	1 (4)
**What are your expectations for the year?**	
Develop teaching skills	22 (79)
Gain PGCert qualification	12 (43)
Develop other skills (not teaching related)	9 (32)
Take part in research	7 (25)
Gain experience for future specialty	5 (18)
Break from clinical work	2 (7)
Normal working hours	2 (7)
Contribute to curriculum	1 (4)
Gain simulation experience	1 (4)
Take specialty exams	1 (4)
**Do you have any concerns about the year?**	
No concerns	10 (36)
Losing clinical skills	5 (18)
Not feeling prepared for role	5 (18)
Difficult student questions	4 (14)
Meeting student expectations	3 (11)
Balancing job with clinical requirements	2 (7)
Dealing with challenging students	1 (4)
Impact on specialty training role	1 (4)
Volume of topics to teach	1 (4)
**How do you think this role will impact upon your future career?**	
Have formal teaching role in chosen specialty	15 (54)
Carry forward teaching skills	9 (32)
Enhanced CV	4 (14)
Beneficial	3 (11)
Help to secure desired specialty role	3 (11)
Possible future career in medical education	2 (7)
Reinvigorate enthusiasm for medicine	1 (4)

#### “Why have you chosen this clinical teaching fellow post?”

All 28 (100%) participants stated they were interested in teaching, 13 (46.4%) indicated they wanted time out of the traditional training pathway, and six (21.4%) said they had chosen a CTF post as they were unsure which specialty to apply for.

Seven participants provided free text answers (NB some participants specified more than one reason) indicating that they had chosen the role because they were changing speciality (*n* = 2), wanted to build their CV (n = 3), wanted to a gain a teaching qualification (n = 1), had been inspired by previous CTFs (n = 1), wanted to build a career in medical education (n = 1), were unsure of where in the country they wanted to live (n = 1), or wanted to work regular hours (n = 1).

#### “What do you think the role will involve?”

Responses were broadly similar to the answers given to the question asking what the key duties were. All expected the role to involve providing teaching, with around one third of participants expecting their role to involve simulation training, planning/organisation of teaching, and/or acting as a supervisor or mentor for students. Individual CTFs were expecting to carry out research or an audit during their year in post (Table 2).

#### “What are your expectations for the year?”

Predominantly the CTFs were expecting to develop their teaching skills, but not all participants stated this. Some listed expectations relating to their future career such as using the year to gain experience for their chosen future specialty while others were expecting the role to provide a break from busy clinical work and allow normal working hours (Table 2).

#### “Do you have any concerns about the year?”

Approximately one third of the CTFs stated they had no concerns regarding the role (Table 2). Concerns mentioned by others included loss of clinical skills, not feeling adequately prepared for the role, and facing difficult student questions. One participant said they were concerned about the impact the role could have on their specialist training role, but no further details were provided to explain what they meant by this. This is a contrast with the participants who said they had chosen the role to build their CVs and those who were expecting to gain experience for future specialties in their answers to previous questions.

#### “How do you think this role will impact upon your future career?”

All thought having held a CTF post would have a beneficial impact upon their future career. The majority (15, 53.6%) were expecting to be able to have a formal teaching component/role in their future chosen specialty (2). Participants also detailed that they thought having held a CTF post would enhance their CV and help to secure a training role in their desired specialty. Three participants stated they thought there would be a ‘beneficial’ career impact, with no further details specified, and one participant answered the question slightly differently by saying they hoped the role would help to reinvigorate their enthusiasm for medicine.

### Focus group

**Fig. 1:**
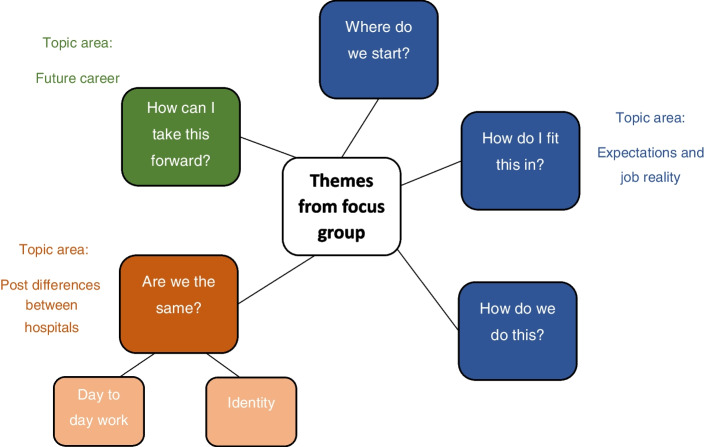
Themes from the focus group and related topic areas

Five main themes were identified. These were; 1) Where do we start?, 2) How do I fit this in?, 3) How do we do this?, 4) Are we the same?, and 5) How can I take this forward?. Broadly, the first three themes map to the topic of expectations and job reality as detailed on the topic guide, the fourth theme (which had two sub themes of ‘day to day work’ and ‘identity’) relates to the topic of post differences between hospitals, and the fifth theme relates to the topic of future career (Fig. 1).

1. Where do we start?

Within this theme, the participants discussed their experiences as they commenced their year in post as a CTF. This included what they had found helpful and where challenges had arisen. There were two aspects to starting their year in post, firstly, the induction they had received to the hospital where they would be working, and secondly, the degree of handover received that was directly relevant to their job.

Some participants said that it had been mandatory for them to attend an induction session(s) at the hospital they worked at, but they had not found these useful as they were not relevant to the job they would be doing. When asked what had happened in terms of induction to their posts, participants reported that they had had to attend inductions for doctors employed to do clinical work.

*Participant 3: “We just sort of turned up? [laughter from group] No, I knew where to go because I've been at that Trust as a medical student so I knew where the CTF office was.”*

*Participant 4: “They made us do the medical induction.”*

*Participant 10: “But it's certainly totally not relevant for us to go to the acute medical clinical induction.”*

When asked in more detail about why they thought that they had been sent to medical induction session, participants reported they felt this was something they had had to do in order to satisfy organisational requirements, and because they were categorised (incorrectly in their opinion) as clinical staff.

*Participant 10: “I think because human resources didn't know what to do with us to be honest. I actually spoke to one and said, Why do I have to go to a medical induction when I’m doing no clinical work? They're like, Oh, well, you've got to go to some inductions [laughter from group]. I thought you'd be more medical that surgical. I was like, well yeah [laughter from group].”*

Whilst these Trust-wide generic inductions were not seen as helpful, one participant described a more CTF-orientated informal induction session that had been beneficial for them.

Participant 4: “To be fair, one thing that they did do with us is they had a representative from each of the main specialities who met with us in the first few weeks to come, it was quite good. It was quite like a question and answers and any questions we had about, you know, how would you advise we teach this aspect of the cardiac exam or whatever it is with a consultant cardiologist. So that was that was actually pretty useful.”

A difficulty with starting the post highlighted by the group was that due to the CTF post only being for one year, there was no overlap between the outgoing and incoming CTFs. This meant there was no formal handover process at the beginning of their jobs, however, several of the participants described circumstantial handovers that had been able to happen in their Trusts due to members of their team having prior CTF experience. These were beneficial in terms of learning how to do practical aspects of the job such as ordering materials or booking rooms for teaching sessions and helping to identify what had/had not worked in previous years.

One participant described how receiving a handover document from the previous group of CTFs was useful, and how one of their colleagues had previously been a CTF so had experience of the practical aspects of the role.

*Participant 4: “The previous CTF team to us wrote a very helpful document, which I mean, they’d just been compiling throughout the year, on like, we did this, it was great, we did this, it was awful, definitely do it differently. And that was really useful. But actually more useful than that, we were quite lucky by chance in that one of our current full time CTFs did it as a four month academic F2 role last year. So he had had first-hand experience of various things, so the first kind of month or so he was kind of guiding the rest of us through the practicalities, because there are differences in how things work on paper versus how things work in practice, and he was really useful for us in terms of practical advice.”*

2. How do I fit this in?

Within this theme, the participants described their experiences of the time pressures they faced as CTFs. These related to the amount of teaching work they were expected to do within their contracted hours, the additional work related to teaching they had to do, and the clinical work that they either wanted or were expected to do (e.g. clinical responsibilities as part of their contract or maintaining clinical skills to avoid deskilling over the year).

Some of the participants discussed how they had to use their own time outside of contracted hours to do preparation work as there was not enough time within their working day to get everything done. One suggested that this was due to the large number of students they were being expected to teach, and perhaps the Trusts did not realise the volume of additional work that was required outside of delivering teaching sessions.

*Participant 9: “I think all of us use a lot of time outside of work to prep for teaching. And that's not taken… there's not enough time in the just, the actual working time to do, to do justice to all the teaching that we do.”*

*Participant 2: “I don’t think they recognise when they employ us, the fact that we’ve got a timetable, to make materials, to prep for teaching sessions, and time for planning and learning things, and whatever you’re doing. It’s a struggle to fit it into a normal amount of time, and they expect you to see so many students.”*

The participants highlighted the importance of taking time to plan and develop sessions. This was felt to be beneficial for both the students as they are receiving high quality teaching sessions, and for the CTFs themselves in terms of teaching skills development. Planning and developing sessions was identified as being time consuming, with insufficient allowance for this in their contracted schedules.

*Participant 4: “I think resources get passed down year to year which is great. And by all means we use them a great deal, we don’t recreate every session. But even that, if you're giving a session, you might have your own way of doing it. And I think it's in the students’ interests and our interests as developing as teachers to not just regurge other people's stuff and to actually put our own spin on it, and like, like you said, that takes quite a considerable amount of time.”*

*Participant 9: “You just can’t use other people’s stuff because you haven't looked through it yet and you end up just reading it.”*

*Participant 4: “Yeah, it's very, it's very obvious having sat in as a student, when that happens, that's being done and you don't get much from it. I think it kind of cheapens our job if that's what we're doing…So yeah, I think there's a disconnect between the time allocated for planning a session or two a week for all the other sessions filled with teaching. I think there's a big disconnect.”*

Despite all being employed as CTFs, there was variation in how much clinical work the participants were expected to do as part of their individual contracts. Some had one day per week allocated to clinical work, some had a half day, and others had no time allocated at all. Regardless of how much time the contracts allocated to clinical work, the primary purpose of the CTF role was to provide teaching and this had to be prioritised over clinical work; with one participant reporting personal development opportunities being sacrificed in order to deliver teaching sessions.

*Participant 8: “With clinical days, for kind of, if you have like a half clinical day, what I found, in the place I work is where you’ve kind of set a date in stone that oh this is the day I'm going to take as a clinical day. But obviously things change during the week or unexpected changes pop up so you might have to run an ad hoc teaching session. And obviously, your role is to look after the students’ education. So you kind of sacrifice, sometimes your own personal development, because you're actually trying to improve the students.”*

Another participant detailed how they have protected time at their Trust for clinical work, but this does not solve the issue of time pressures as it generates more work, and instead carrying out additional locum shifts was a way of keeping clinical skills practised over the year.

*Participant 6: “Some discussions about how can we have a clinical time where we work at [Trust name] is that, yes, we have the kind of protective, protected afternoon to do the clinical stuff. But actually, if you do the clinical stuff, you’re really busy, that's only putting more work on yourself and you might as well just get the clinical skills if you do a four hour locum shift in the evening and that’s what people have tended to have been doing.”*

Conversely though, a third participant reported that they did have enough time for clinical work, highlighting the variation between CTF jobs and Trusts.

*Participant 5: “I think there’s a lot of difference between the different roles at different trusts in different areas. Like for example, we do have a clinical day where I was supernumerary but we can basically choose where we spend that time essentially.”*

3. How do we do this?

Participants described the challenges and difficulties they faced in meeting the requirements of the role. These were broadly split into shared difficulties that were encountered by several CTFs across different Trusts, and individual difficulties that were post or Trust specific.

The shared difficulties reported by the participants fell into three main categories; a) continuity issues caused by the short length of post, b) large student numbers, and c) lack of standardisation regarding teaching content.

- Continuity issues caused by the short length of post

A difficulty with the role was reported as being not being able to make changes to practice and assess any improvement that these might have. As the CTF posts are only a year in length, and without any guaranteed formal handover as discussed in a previous theme, those who implement any changes are unable to evaluate them. Whilst there are senior staff who are in posts of longer duration, as they are not directly involved with the delivery of teaching, they are not able to fully appreciate any challenges that might need addressing. One participant suggested that having a CTF employed on a permanent basis might help to solve this problem.

*Participant 6: “I think is quite difficult that everybody moves on, like, the posts are usually only a year post, and you can extend it kind of by mutual agreement, but there's, you know, the seniors are obviously the continuity, but unless you're delivering the teaching on the ground, you don't fully understand the difficulties and what's going on. I don't know if they'll be able to recruit a permanent CTF, that's kind of a continuity. And they can think about this didn't work very well last year, let's try this. You can never, if you're just working for a year, you can never kind of close the loop and improve something because it gets to audit or tick boxes you’re not there to see the benefit or to evaluate it and see whether it’s working or not.”*

b) Large student numbers

Participants described difficulties arising from the large number of students they were being expected to teach, both on an individual teaching session level and over the year as a whole. Participants described not being able to find rooms large enough for all the students or having to run sessions with more students that they would have liked.

Participant 6: “There was quite a large cohort of particularly the fourth year students, and in terms of running [a] simulation we ended up with what 15–20 people? And it just didn't work”.

Some participants felt the Trusts had not adapted to manage increased student numbers, and this put pressure on CTFs in terms of time and how many students they were expected to teach. The impression given was that the Trusts should have considered the impact of increasing student numbers and made changes either by structuring jobs differently or by hiring additional CTFs.

Participant 3: “I'm not even sure if the Trust is that proactive about planning ahead, sometimes. So like [Trust name] took on 18 more 3rd years, but the same number of CTF, like, we'll just do it like we did it last year, even though in fact, what you're now got four more firms or whatever. But it's like, well, we'll still use Iast year’s timetable and we’ll still schedule everything in the same way. We'll just squash the timings down a little bit and I'm sure it will be fine.”

c) Lack of standardisation regarding teaching content

Participants described difficulties with knowing what to teach students, particularly the third year students. The main issue with this year group was there was not the same level of guidance provided from the medical school as for other year groups (e.g. participants’ detailed handbooks and specific learning outcomes for fourth and fifth years), and as a result, different Trusts would teach things in different orders. This meant for the CTFs, when they were teaching students in the second semester who had been at different Trusts for the first semester, they had a mixture of students who had been taught certain things already and those who had not, and therefore it was difficult to plan sessions accordingly.

*Participant 9: “Like their clinical skills, and then that booklet is really quite descriptive of what they can do each semester. And that can give you quite a bit of guidance, particularly, particularly for third years. But I do feel like different Trusts have like completely different ideas about which ones they'll introduce into in the semester early. And so like cannulation, our Trust decided we're going to start my teaching foundation as soon as they come for their clinical rotations, whereas there was a few that came in semester two that had not done it at all. Again, that kind of situation where you're teaching people who've got loads of experience and people who’ve got absolutely none. Very difficult to pitch your session.”*

It was acknowledged that knowledge will vary as a result of what students have been exposed to on their placements, but the participants felt that it would be helpful for some parts of the course, e.g. clinical examinations, to be standardised to avoid having to repeat teaching to some who had already been taught.

*Participant 3: “With the 3rd years, 3rd year is a bit of a waffle-y year, sort of no continuation throughout, I think there are some times you'll have some of the new cohort students after Christmas, you ask them, for example, who's learnt a knee exam, and like some hospitals will have done it, some haven’t, and that's gonna be the same problem, then even within a firm, one of them may have done it, the other three haven't. So then you end up repeating a lot of stuff, particularly the examinations.*

#### Individual difficulties

Individual difficulties mentioned by participants related to a part-time working pattern being challenging, and lack of local knowledge. One of the participants who worked part-time in a job share said they had felt pressure from missing out on things that happened on their non-working days and wondered how their employer really felt about having two people sharing one post.

*Participant 6: “I’ve been doing the role less than full time and that's been quite difficult. Juggling kind of a job share has been quite difficult. I’m feeling the pressure of missing out on team meetings and stuff that fall on my non-working days and trying to juggle childcare is then a lot more difficult than what I was anticipating actually. They were kind of very flexible and open about at the beginning, but now being honest, I do wonder if my employer thinks, oh, actually, this is quite an inconvenience having two people sharing the role and logistics, I don’t know, that just might be my inference.”*

One of the participants had not studied at Birmingham medical school and therefore did not have the same ‘local knowledge’ as other CTFs did about what the students would have already known ahead of their clinical placements. They reported that this made initial planning of teaching sessions difficult.

*Participant 7: “I find it quite difficult because I'm not a Birmingham med student as well. So at the beginning, I have no idea what they know, what they don't know if I didn't, so in our office the rest of us were Birmingham med students at [Trust name] and, so it's really, you kind of had a month you're supposed to prep stuff. And I had no idea what I was prepping, I didn't know how much they knew, how much they didn't know, having some more guidance on that would have, I would have found it helpful but that I then kind of picked up from someone who'd been to Birmingham.”*

4. Are we the same?

Within this theme, two subthemes were identified. The first, ‘day to day work’, covered participants discovering and discussing differences between their own posts and experiences, and the second, ‘identity’, covered how they as CTFs were seen by other people present in their work environment.

#### ‘Day to day work’

Key differences in the posts and way that CTFs had to work in their Trusts seemed to be determined by what participants felt were the Trusts’ priorities and the numbers of students they were required to teach. Some participants felt that their Trusts prioritised the number of students that could be taught/sessions delivered over the quality of the teaching itself.

*Participant 3: “I think just following on from some of those points, there is sometimes an emphasis on quantity, not quality [audible agreement from several participants], that the Trust is very much like ah we can then say, to med school, or whatever, we've given them three hours of surgical teaching this week, and that brilliant, or our CTFs are doing all of this. Whereas sometimes in reality, I think, if I did half as much teaching, I could prepare it twice as well. And it would actually be better. But then the Trust, it doesn't look as good on paper when they say Oh, our CTFs only giving them, each firm, two hours of teaching a week versus four.”*

But as the discussion went on, there was a participant from another Trust who had had a very different experience in their post, which seemed to be due to smaller numbers of students. The other participants seemed surprised to hear about how different this post was to their own.

*Participant 8: “I don't think it's the same in every Trust, because I work in [Trust name] and we don’t have very many students. We’ve got the 5th years and in charge of them there’s 4 CTFs [laughter and gasps from several participants]. Not just for the 5th years but across the years so in terms of, we can quite easily give them all simulation on one afternoon. And kind of run a tutorial several times in small groups, but it only takes a morning.”*

*[pause]*

*Facilitator: “You look quite taken aback by that.”*

*Participant 6: “I just wonder how the ratio of students to CTF gets worked out [agreement noises], because that sounds really nice and what it should be [laughter] but it’s not the case where we work.”*

### ‘Identity’

This subtheme covered participants’ views on how the other people in the hospital where they worked (doctors, patients, non-medical staff) interacted with them. Participants felt that for patients it could be confusing interacting with a CTF because whilst CTFs are doctors, they have different duties to the majority of doctors in the hospital. One participant described the importance of clearly explaining to patients what their role was and what difficulties can arise.

*Participant 8: “When you go into the wards, I always introduce myself and just say, this is who I am. When you explain it to patients, that can be a bit difficult, because they'll see you as a doctor, I'm wearing a stethoscope, so they think you're in charge of their care. And you have to make it clear that I'm not your responsible doctor.…. So you have to then signpost them to the team looking after them. So that's some of the difficulties I found with role identity. Obviously, I'm one of the teaching doctors, I look after the medical students, probably the way I kind of approach it.”*

The participants reported having good working relationships with the other doctors based at the hospital, and these relationships were mutually beneficial for both parties. The participants described being reliant on other doctors to assist with finding patients suitable for teaching sessions, which other doctors in the hospital were willing to do.

*Participant 9: “I found that actually other doctors have been quite understanding, they seem to recognise the role and they know you're looking for patients to bring students to and they’re actually quite helpful.*

*Participant 2: “I think most people almost as well, there are students around, we do, it's like there’s teaching all the time. And most of the consultants are involved in teaching sessions as well. So they are kind of aware that we're doing this. And once you go on a ward, it's not my patients so I’ll ask the F1, I want to teach on that do you have any patients, or the nursing stuff and get help from that way as well, to find the actual patients, so find it's always, they’re always very helpful.”*

They also described depending on the help of doctors to help deliver teaching sessions, with one participant describing a successful recruiting scheme that had been put in place by a predecessor.

*Participant 4: “We rely quite heavily actually, because of the number of students. Lots of our sessions just need, like people to facilitate stations. So we have at least twice a week, for every week, pretty much like a signup sheet, where we ask for doctors available, that tends to be kind of the more junior F1 F2 is signing up. And I was actually really surprised that we get the people signing up, they do, I thought that we'd really struggle. But again, this guy did it last year so now it tends to be pretty good and we get multiple doctors coming every week, who are pretty engaged in teaching.”*

For the other doctors wanting to get involved with teaching, working alongside the CTFs was beneficial in terms of gaining evidence of teaching experience that could be used for job applications. Participants also reported that other doctors were interested in the CTF role itself and finding out whether the job might be something they would want to do in the future.

*Participant 4: “And again, like you said, they're quite interested in the role. And some of them are thinking about doing it. So we can kind of advise on that. So there's quite a good relationship, I think with the CTF team, and particularly the juniors but also the seniors are very helpful as well, when you got that relationship.”*

5. How can I take this forward?

Within this theme, participants discussed their enjoyment of the post despite the challenges and how some wished there was a way of continuing their career in a similar role.

The participants described having benefited from the year in terms of their own learning and the job being one they would recommend. One participant commented that other doctors had been interested in what the CTF job was like as they were giving the impression that it was a really positive thing to be doing.

*Participant 4: “I just know we've been discussing a lot of challenges, I know that was more the focus but I've also absolutely loved it, I'd hands down, 100% recommend it to anyone that asked, so that would be, even though I’ve been churning out all these challenges and stuff that my overall take would definitely be one of really enjoyed it got a lot out of it and 100% recommend it so yeah.”*

*Participant 7: “So many doctors have said what do you do? How do I get involved with this? It sounds like you're giving off a positive attitude, looks like you’re enjoying your job.”*

One participant expressed a desire for a way to continue the role in a formal capacity which others agreed with. There was acknowledgement that there are some jobs like that available, but they are not readily or easily available.

*Participant 5: “One thing I do wish is that it was kind of like a more like formal training I know you can't make a training programme of it but like whether it was like part of, there was some sort of way that we can keep it up through core training in some way shape or form but in a more formal way [agreement from other participants]”.*

*Participant 6: “Like an academic training pathway but specifically for students.”*

*Participant 5: “I think it does exist, there are a few posts like that, but if it was more of a sensible way of doing it. That would be good.”*

## Discussion

This study has allowed the first exploration of the expectations of CTFs prior at the beginning of their role and their experiences at a mid-year point in their year in post. The results have given an insight into some of the challenges faced by the CTFs particularly with regards to difficulties starting the role and the time pressures they faced. Despite the challenges of the role however, the participants were enjoying their year in post and felt it was a job they would recommend to others. The main findings of the study are discussed in detail below.

Supporting existing literature, all the participants indicated in the baseline survey they had chosen a CTF post because they were interested in teaching and/or wanted time out of training pathway and/or unsure of which specialty to choose [14, 17, 28]. The free text answers to this question suggested that a CTF post is perceived to have benefits beyond gaining teaching experience, with participants detailing they had chosen the post to enhance their CV and gain extra credit for future job applications. This is particularly relevant to junior doctors currently with growing numbers of applications for specialty training posts increasing the competition to secure such a post and enable career progression [29], and is likely to be a reason why a CTF post is currently a desirable job for junior doctors.

A large proportion of participants indicated in the survey they had chosen the post as they wanted time out of the training pathway. A recent report by Health Education England (HEE) has shown that the number of doctors taking a break from training after completing the first two years after qualifying has risen from 17% in 2010 to 65% in 2019 [30]. Reasons given in the report for wanting to take a break were wanting time out from the pressures of a training pathway, being unsure of which specialty to commit to, feeling burnt out, wanting more control over their time, and wanting to take other opportunities (both professional and personal). Although some participants in this study were beyond two years after qualification, most participants were at two years post qualification, and offer similar reasons for choosing a CTF post in this study to those given for taking a break from training in the HEE report. A CTF job may be seen as a particularly advantageous role for someone wanting a break, as in addition to offering a year out of training, it offers development of teaching skills and the opportunity to gain a post-graduate qualification.

The focus group has given an insight into some of the challenges faced by the CTFs with regards to difficulties starting the role and time pressures faced. A key difficulty identified by the participants was a lack of formal handover and appropriate Trust induction. As the CTF role is not part of a hospital-based training pathway, it does not have the usual structure of trainees at different levels who would be able to provide a handover and is a stand-alone year post meaning there is no overlap between the incoming and outgoing post holders. The participants who did receive an informal handover either benefitted from a colleague with prior CTF experience or, in one case, an organised schedule of meetings with senior colleagues from various specialties. A key area of uncertainty was around what medical students in each year needed to be taught. It could be beneficial for Trusts/those employing CTFs to consider how more formal handover processes could be put in place for new CTFs to try to eliminate some of the challenges experienced at the start of a new post.

With the CTF post still being a relatively new job across many hospitals, it may be that the need for a separate induction has not yet become apparent to senior and organisational staff. It is interesting to note however that whilst the participants in this study felt it had not been relevant for them to attend the induction for clinical staff, most of the participants did carry out clinical work through either being contracted to or through taking on additional locum shifts, suggesting some form of clinical induction was appropriate. This could be to do with role identity, with the CTFs themselves identifying as a different type of doctor (for students, rather than patients), or viewing the clinical work they provide as different (e.g. locum shifts to learn more about a speciality as personal development rather than usual service provision). With the CTF role being primarily education and student focused, it could be that a desire for an induction/handover relevant to those new and unfamiliar aspects of the role was given greater importance by the participants than an induction relevant to the clinical aspects of the role that participants were already familiar with.

Related to this was the continuity issues caused by short post length, meaning it was difficult for any changes to be made or acted upon. This supports previous findings that rapid turnover of CTF posts can limit the continuity and sustainability of the role, and mean that each year the post holders are starting with limited feedback or information regarding the role [10]. This could be suggestive of a need for those with more seniority and overall responsibility for undergraduate medical education in Trusts to take an active role in overseeing, implementing, and evaluating suggested changes and/or a formal handover. With the CTF role a popular and desirable job, this is something that should be considered to ensure that post holders, Trusts, and medical students get the most out of the role.

A significant challenge for participants was that of time pressures, and the difficulties of fitting the demands of the job into contracted hours. This impacted upon both contracted duties and personal development. For the participants, there seemed to be a disconnect between what they were expected to deliver in terms of teaching and planning, and what was possible with the time available and number of students to teach. This was related to a sense that some Trusts were not reactive to increased student numbers and had prioritised volume of teaching delivered over quality. It could be that as the CTF post is still relatively new, the Trust management teams are still working out how best to utilise CTFs and their time, or that the CTF posts were created to manage teaching pressures in a particular environment that has since changed due to increasing student numbers and there is a lack of proactivity in assessing and adapting the CTF role and duties to the current needs. Having people in posts that are primarily responsible for education differs from the traditional model of delivering medical education where teaching sessions were delivered on a more ad hoc basis by senior doctors primarily employed in clinical roles. Time pressures are known to also be a challenge for consultants who teach [31] and having CTFs available to deliver teaching relieves some of these pressures for senior doctors. It is interesting to note though that the participants in this study reported themselves relying on other doctors to assist with teaching sessions to compensate for the time pressures they themselves were facing. It could be that for some Trusts, CTFs alone are no longer sufficient to deliver the volume of teaching required, but this study was not able to explore reasons that could be behind this such as whether these Trusts had had a particular increase in the number of students they hosted or if they had decided to increase the amount of teaching delivered. Whilst these time pressures are something that may always be an issue for hospital-based teaching, further evaluation of the CTF role and duties may be beneficial in relieving some pressures and optimising the quality of teaching delivered by CTFs. Previous work has suggested that CTF posts should have dedicated time for teaching, administrative work, and clinical work, with these three aspects being given equal importance [10], and future work should build on exploring optimising CTF posts from the point of view of a variety of stakeholders in medical education. It could also be that time pressures are a particular issue at the start of CTF posts due to the relative inexperience of those hired to fulfil teaching roles. Posts could be adapted with more time allocated for planning at the start of the role to take into account inexperience and unfamiliarity with teaching and the associated planning required.

With CTFs’ personal development being sacrificed in order to deliver teaching and relieve time pressures on senior doctors, the question of whether the CTF posts are primarily service provision posts or training posts is raised. It could be that the posts are seen as having different primary functions by different stakeholders, but it is likely that the CTFs view the posts as a training post, with the expectation of development and opportunity to gain a postgraduate qualification offered within these roles [7]. It was evident from the baseline survey that the CTFs in this study had the expectations of opportunities outside of teaching such as participation in research, development of other non-teaching related skills, and gaining experience in future desired specialties, but that as revealed in the focus group, teaching was having to take priority over other opportunities. Future research exploring expectations and realities of a CTF post would be beneficial. It could be that tensions between expectations/function of the role are a contributory factor in the challenges perceived in the role, and work exploring this would be beneficial to optimise the role.

A further challenge was the lack of standardised teaching across Trusts in terms of what students were taught in each semester at each individual Trust. For the participants, this made planning sessions later in the year difficult as students had been exposed to different teaching on their previous placement. This could also make students’ learning less efficient as some students will cover the same topics twice or may miss being taught others completely. The participants’ suggestion of some kind of standardisation for particularly the third year students seems like a sensible step to take to address this and to improve the efficient of teaching and learning. Further consultation work between the Trusts, the medical school, and the CTFs may be beneficial for areas such as this.

Individual difficulties mentioned in this study were a part-time working pattern being challenging and not having ‘local’ knowledge through not being a UoB graduate. Regarding ‘local’ knowledge, the implied expectation that CTFs will know what the medical students need to be taught as they have studied at the same university and the reported lack of formal handover could make starting the role very difficult for CTFs that have studied in a different region. Further work is merited to identify whether this is an issue faced by other CTFs.

It is interesting that concerns highlighted in the baseline survey such as facing difficult student questions and losing clinical skills were not discussed in the focus group. It could be that the concerns at the beginning of the post did not turn out to be issues when actually doing the role or that other challenges were more of an issue.

As supported by other studies, the participants reported variation between their posts in terms of duties and experiences [10, 14]. Whilst participants were aware of some differences e.g. amount of time contracted to clinical work, there was one particular difference, the small student numbers at one individual Trust, that took participants by surprise. One participant commenting that the smaller student numbers sounded ‘nice and how it should be’ whilst acknowledging that it was not like that where they worked, particularly highlighted the different experiences CTFs have across their posts.

A previous study found that CTFs experienced stigma towards the role from other clinicians [10], however, this study has found the opposite with participants reporting good working and mutually beneficial relationships with other doctors. This is suggestive of the CTF role being well integrated in the Trusts with other doctors being willing to help with teaching and identifying suitable patients, and also interested in finding out whether this was a job they would like to do.

The majority of participants expected to have a formal teaching role in their future career, suggesting holding a CTF post could be predictive of who will go on to be medical educators in the future, and there was a clear desire to continue a career in a similar role or for there to be a more formal training pathway. With the increase in number of CTF posts available, this could indicate more doctors are hoping to pursue a career in medical education and demonstrates a growing interest in medical education as a speciality. However, it has been noted that medical education is becoming increasingly formalised [32], and coupled with the General Medical Council’s requirement that all doctors should be prepared to contribute to the teaching and training of doctors and medical students [33], it could be that interest in medical education is simply being recognised more than before.

As the participants said in the focus group, there are only a few jobs in medical education available, and this echoes findings of a previous study suggesting there are uncertainties in how to pursue a career in medical education with only limited support available [10]. With many doctors opting to work in medical education for a year as a CTF, consideration should be given to how to support the development needs of those interested in pursuing a medical education career. Some relevant initiatives have already begun, for example, the NIHR Incubator for Clinical Education Research [34], which aims to develop clinical education research as an academic field. Whilst this particular initiative is aimed at those who want to conduct educational research in a clinical setting, this could still be of interest to those who have held a CTF post and help to support career development in this field.

### Strength and limitations

A strength of this study was the sampling strategy used to identify and recruit participants. As CTFs in the West Midlands work at a number of individual Trusts, recruiting them from a single site (UoB, where they are offered the opportunity to register on the PGCert course) was efficient. As no central register is kept of all those employed as CTFs and CTFs are employed directly by Trusts, it would have been difficult to find and approach individual CTFs. Additionally, typically CTFs stay in post for one year but some may stay on for a second year as was found in the results of this study. If this was the case they would not register again for the PGCert course, meaning that recruiting from UoB allowed for only new-to-post CTFs to be invited to participate. It would not have been appropriate to include CTFs who had already spent time in post in the study as the aims included exploring role expectations prior to commencing the post.

An additional strength of this study was the willingness of participants to take part and discuss their experiences in a focus group. Over half of the participants recruited to the study indicated they were willing to take part in a focus group, and all those who did participate contributed to the discussion. Only minimal facilitation and prompting was required from the research team during the group as the participants responded to each other sharing their own experiences.

A further strength of this study is its high degree of trustworthiness, assessed by evaluating the methods of this study against the four components of trustworthiness as proposed by Lincoln and Guba [35]: credibility, transferability, dependability and confirmability. Credibility has been achieved in this study by following rigorous methods for gathering and analysing data. The data was analysed following Braun and Clarke’s steps for thematic analysis [26], and regular meetings were held with the research team discuss the coding and analysis of the transcript. Transferability has been achieved by providing as much detail about the participants as possible whilst maintaining confidentiality. Dependability has been achieved by explicitly reporting methods for each stage of the study clearly, and other members of the research team were involved in the analysis of the data as described above. Confirmability can be said to have been achieved when credibility, transferability, and dependability have been achieved [36].

There are however some limitations to this study.

Firstly, not all of the participants took part in the focus group. This means the results may not be representative of the views of the wider population in the study who took part in the baseline survey but did not take part in the focus group. Due to the COVID-19 pandemic a second focus group was unable to be held as planned. Available literature suggests that at least 80% of themes on a topic can be captured with two to three focus groups using a semi-structured guide [37]. Therefore, it is unlikely that data saturation was reached through the single focus group that took place. Despite this, this exploratory focus group provided a large amount of data, and the analysis has revealed a number of themes providing an insight into the experiences of this group which aligns with existing literature.

Secondly, it is known that focus groups can result in participants not wishing to share views that they perceive differ from the majority of the group [38]. It is unclear whether this was an issue with this focus group, however participants appeared to be willing to share their individual experiences as part of the discussion.

Thirdly, whilst the participants in this focus group were from a selection of Trusts across the West Midlands region, this still presents a geographical limitation. However, as medical education in the UK is regulated by the General Medical Council and delivered with the NHS (a national level service), it is likely that these results are applicable to other areas of the UK and potentially to other countries with similar models of delivering undergraduate medical education. This work therefore may be useful to medical education decision makers in the West Midlands region and beyond, as well as providing a starting point for future research on a national scale regarding the experiences of CTF post holders.

## Conclusion

This study has added value to the existing limited literature which has not explored CTFs’ views and expectations prior to being in post. Findings about why doctors decide to take up a CTF post including both career-driven reasons and lifestyle-driven reasons are useful to help understand why increasing numbers of doctors are electing to spend a time in a CTF post. This study is suggestive of today’s workforce of junior doctors being willing to step outside of the traditional hospital doctor career route to spend time on interests that have not previously been possible in a usual career trajectory, and the value of work-life balance in a medical career.

This research may be of use to anyone who employs CTFs in a hospital setting and wishes to consider how to best utilise CTFs or how to refine the role to ensure job satisfaction and meeting of expectations. Key suggestions for those employing CTFs as identified by this study include ensuring there is a formal handover process in place between outgoing and incoming CTFs. It is also important that there is someone at each Trust with responsibility for evaluating changes suggested by CTFs and it would be worthwhile for there to be central co-ordination of medical education stakeholders in regions that have multiple hospitals hosting students from the same medical school. Consideration should be given whether posts could be structured with more planning time at the start to relieve time pressures on CTFs who are inexperienced in planning teaching sessions. The balance of contractual duties and personal development time in posts offered also requires consideration.

As the CTF role is growing in popularity and numbers, and increasingly relied on for the delivery of undergraduate medical education, consideration of the challenges facing doctors employed in this role is crucial. By understanding the experiences of post holders and an appreciation of difficulties in the job, changes to practice could be made to ensure that CTFs get the most out of the year in post, and the medical education they deliver is optimised. Future research exploring the CTF role in more depth and on an individual level through in-depth interviews, and in other areas of the country would be beneficial to identify shared challenges and potential solutions to problems.

### Supplementary Information

**Supplementary Material 1.**

**Supplementary Material 2.**

## Data Availability

The datasets generated and/or analysed during the current study are not publicly available due to participant confidentiality but are available from the corresponding author on reasonable request.

## References

[1] Medical Schools Council. The Expansion of Medical Student Numbers in the United Kingdom. Medical Schools Council Position Paper. October 2021. Available From: https://www.medschools.ac.uk/Our-Work/the-Expansion-of-Medical-Student-Numbers Accessed 20 Mar 2023.

[2] NHS England. NHS Long Term Workforce Plan. 2023. Available From: https://www.england.nhs.uk/Publication/Nhs-Long-Term-Workforce-Plan/. Accessed 5 July 2023.

[3] Department of Health. The NHS Plan. A Plan for Investment. A Plan for Reform. London: Stationery Office 2000.

[4] Hendry RG (2005). Consultant attitudes to undertaking undergraduate teaching duties: perspectives from hospitals serving a large medical school. Med Educ.

[5] General Medical Council. Tomorrow's Doctors: Recommendations on Undergradaute Medical Education. 2002.

[6] Darragh L, Baker R, Kirk S (2015). Teaching medical students, what do consultants think?. Ulster Med J.

[7] Marriott C, Boyd J (2020). Clinical teaching fellows: a mutually beneficial relationship. Trends in Urology & Men's Health.

[8] Couchman D, Donnachie D, Tarr J, Bull S (2022). clinical teaching fellows, the new norm?—experiences of fellows and education faculty. Clin Teach.

[9] Chu A, Morton C, Pye C, Ghani L, Smith SF (2019). Clinical teaching fellowships – enhancing the out of programme experience through a peer network. Clin Med.

[10] Ker R, Guckian J, Bowey AJ. ‘Just a Year Out’? – Challenges of the Clinical Teaching Fellow. MedEdPublish 2018;7.

[11] Wilson S, Denison AR, McKenzie H (2008). A survey of clinical teaching fellowships in Uk Medical Schools. Med Educ.

[12] van Heerden C, Uahwatanasakul W, Vaughan B, Delany C (2020). Ripple effect of a clinical teaching fellow programme in an Australian paediatric hospital. J Paediatr Child Health.

[13] Brown C (2015). Clinical teaching fellows: best of both worlds?. Clin Teach.

[14] Campbell S, Cifelli P (2018). So you want to be a medical education fellow. Ulster Med J.

[15] Fisher J, Smith S (2016). Confessions of a clinical teaching fellow. Clin Teach.

[16] Furmedge D, Verma A, Iwata K (2013). The rise of clinical teaching fellowships. BMJ.

[17] Pippard B, Anyiam O (2016). The many roles of a clinical teaching fellow. BMJ.

[18] Rimmer A (2019). How do I select the right clinical teaching fellowship?. BMJ.

[19] Woodfield G, O'Sullivan M (2014). Clinical teaching fellows: everyone's a winner. Clin Teach.

[20] Harris IM, Greenfield S, Ward DJ, Sitch AJ, Parry J (2023). The clinical teaching fellow role: views of the heads of academy in the West Midlands. BMC Med Educ.

[21] Kitzinger J (1995). Qualitative research: introducing focus groups. BMJ.

[22] Vaughn S, Shay Schumm J, Sinagub J (1996). Focus Group Interviews in Education And psychology.

[23] Krueger R CM. Focus Groups a Practical Guide for Applied Research. 5th ed. Thousand Oaks, California: SAGE Publications, Inc; 2014.

[24] Braun V, Clarke V. Thematic Analysis. Apa Handbook of Research Methods in Psychology, Vol 2: Research Designs: Quantitative, Qualitative, Neuropsychological, and Biological. Apa Handbooks in Psychology®. Washington, DC, US: American Psychological Association; 2012:57–71.

[25] Kiger ME, Varpio L. Thematic Analysis of Qualitative Data: Amee Guide No. 131. Med Teach. 2020;42:846–54. 10.1080/0142159X.2020.1755030 32356468

[26] Braun V, Clarke V (2006). Using thematic analysis in psychology. Qual Res Psychol.

[27] Qsr International Pty Ltd. (2020) Nvivo 12 Plus (Released March 2020), https://www.qsrinternational.com/Nvivo-Qualitative-Data-Analysis-Software/Home.

[28] Thomson R, Loveland A, Stewart J, Fisher J (2016). How to stop the runaway train of clinical training. BMJ.

[29] Torjesen I (2022). Specialty training: record number of applicants and posts filled in England. Say Officials BMJ.

[30] Health Education England. The F3 Phenomenon: Exploring a New Norm and Its Implications. February 2022. Available From: https://www.hee.nhs.uk/Sites/Default/Files/Documents/F3_Phenomenon_Final.Pdf Accessed 13 May 2022.

[31] Harris IM, McNeilly H, Benamer H, Ward DJ, Sitch AJ, Parry J (2021). Factors affecting consultant attitudes to undertaking undergraduate medical student teaching in the Uk: A systematic review. BMJ Open.

[32] Mulholland M, McNaughten B, Bourke T (2022). ‘I'm a Doctor, Not a Teacher’: The roles and responsibilities of paediatricians in relation to education. Arch Dis Childhood Educ Pract.

[33] General Medical Council (2014). Good Medical Practice.

[34] National Institute for Health and Care Research. Clinical Education Incubator. March 2021. Available From: https://www.nihr.ac.uk/Documents/Clinical-Education-Incubator/24887 Accessed 24 Feb 2023.

[35] Lincoln Y, Guba EG (1985). Naturalistic Inquiry.

[36] Thomas E, Magilvy JK (2011). Qualitative rigor or research validity in qualitative research. J Spec Pediatr Nurs.

[37] Guest G, Namey E, McKenna K (2017). How many focus groups are enough? building an evidence base for nonprobability sample sizes. Field Methods.

[38] Smithson J (2000). Using and analysing focus groups: limitations and possibilities. Int J Soc Res Methodol.

