# Supplementary material for: The Clinical Teaching Fellow role: exploring expectations and experiences
Source: BMC Med Educ. 2024 Mar 1;24:213. doi: 10.1186/s12909-024-05207-6 (PMC10908057; doi:10.1186/s12909-024-05207-6)
Supplement: Supplementary file 1 — Supplementary Material 1. [file 12909_2024_5207_MOESM1_ESM.docx]

**Appendix 1**

**BASELINE SURVEY**

| The first section of this survey will ask you some questions about yourself and your academic history. | | | | |
| --- | --- | --- | --- | --- |
| 1 | What is your date of birth? | | DD/MM/YY | |
| 2 | What is your gender? | | - Female - Male - Other (specify) ____________ | |
| 3 | What is your GMC number? | |  | |
| 4 | What is your ethnicity? | | White   - English / Welsh / Scottish / Northern Irish / British - Irish - Gypsy or Irish Traveller - Any other White background, please describe___________   Mixed   - White and Black Caribbean - White and Black African - White and Asian - Any other Mixed / Multiple ethnic background, please describe   ____________________  Black   - African - Caribbean - Any other Black / African / Caribbean background, please describe___________________   Asian   - Indian - Pakistani - Bangladeshi - Chinese - Any other Asian background, please describe_____________   Other   - Arab - Any other ethnic group, please describe_____________ | |
| 5 | Which medical school did you attend? | |  | |
| 6a | Which MBChB course did you complete? | | - Undergraduate - Graduate entry | |
| 6b | What year did you qualify with your medical degree? | |  | |
| 7 | Did you have a degree prior to commencing the MBChB course? | | - Yes – go to question 7a - No – go to question 8 | |
| 7a | Please provide details of your previous degree | | University:  Subject:  Date:  Class: | |
| 8 | Did you complete an intercalated degree whilst at medical school? | | - Yes – go to question 8a - No – go to question 9 | |
| 8a | Please provide details of your intercalated degree | | University:  Subject:  Date:  Class: | |
| 9 | Please list the posts you held each year **before** your CTF post | | | |
|  | **Date**  **From** | **Date**  **To** | | **Post** |
|  |  |  | |  |
|  |  |  | |  |
|  |  |  | |  |
|  |  |  | |  |
|  |  |  | |  |
|  |  |  | |  |
|  |  |  | |  |
| 10 | Do you have any teaching qualifications? | | | |
|  | **Qualification** | **Institution** | | **Date** |
|  |  |  | |  |
|  |  |  | |  |
|  |  |  | |  |
| The next section of the survey will ask you some questions about your CTF post. | | | | |
| 11 | Where is your CTF job? | |  | |
| 12 | How long is the post? | |  | |
| 13 | Is this full time or part time? | | - Full time - Part time | |
| 14 | What will your key duties be? | |  | |
| 15 | Who are your named supervisors? | |  | |
| The next section of the survey will ask you some questions about why you chose to do a CTF job and your expectations of the role. | | | | |
| 16 | Why have you chosen this clinical teaching fellow post? | | - Interested in teaching - Want time out of training pathway - Unsure of specialty to choose - Other (specify) ____________ | |
| 17 | What do you think the role will involve? | |  | |
| 18 | What are your expectations for the year? | |  | |
| 19 | Do you have any concerns about the year? | |  | |
| 20 | How do you think this role will impact upon your future career? | |  | |

Thank you for completing this survey.
