# Supplementary material for: The Clinical Teaching Fellow role: exploring expectations and experiences
Source: BMC Med Educ. 2024 Mar 1;24:213. doi: 10.1186/s12909-024-05207-6 (PMC10908057; doi:10.1186/s12909-024-05207-6)
Supplement: Supplementary file 2 — Supplementary Material 2. [file 12909_2024_5207_MOESM2_ESM.docx]

**APPENDIX 2**

Focus group topic guide

- Role expectations/concern

From survey:

- Expectations = developing teaching skills, gain experience for future job
- Concerns = losing clinical skills, difficult student questions
- Reality of job

Matching job description/unexpected aspects/support in role

Managing clinical/educational parts of role

- Post differences from hospital to hospital
- Career development
